# Supplementary material for: A comparative genomics study of 23 Aspergillus species from section Flavi
Source: Nat Commun. 2020 Feb 27;11:1106. doi: 10.1038/s41467-019-14051-y (PMC7046712; doi:10.1038/s41467-019-14051-y)
Supplement: Supplementary file 1 — Supplementary Information [file 41467_2019_14051_MOESM1_ESM.pdf]

Supplementary information for article:

‘A comparative genomics study of 23 Aspergillus species from section Flavi’ by Kjærbølling et al.

Supplementary Table 1 - Overview of species and genomes used in this study [3,12,14,17,21,24,25,37,88–91].

Genus	species name	Section	JGI portal	References
Aspergillus	*campestris*	*Candidi*	genome.jgi.doe.gov/Aspcam1	[17]
Aspergillus	*steynii*	*Circumdati*	genome.jgi.doe.gov/Aspste1	[17]
Aspergillus	*coremiiformis*	*Flavi*	genome.jgi.doe.gov/Aspcor1	This study
Aspergillus	*pseudocaelatus*	*Flavi*	genome.jgi.doe.gov/Asppsec1	This study
Aspergillus	*pseudonomius*	*Flavi*	genome.jgi.doe.gov/Asppsen1	This study
Aspergillus	*avenaceus*	*Flavi*	genome.jgi.doe.gov/Aspave1	This study
Aspergillus	*bertholletius*	*Flavi*	genome.jgi.doe.gov/Aspber1	This study
Aspergillus	*caelatus*	*Flavi*	genome.jgi.doe.gov/Aspcae1	This study
Aspergillus	*flavus*	*Flavi*	genome.jgi.doe.gov/Aspfl1	[13]
Aspergillus	*fumigatus*	*Fumigati*	genome.jgi.doe.gov/Aspfu1	[37]
Aspergillus	*nidulans*	*Nidulantes*	genome.jgi.doe.gov/Aspnid1	[21]
Aspergillus	*niger ATCC 1015*	*Nigri*	genome.jgi.doe.gov/Aspni7	[89]
Aspergillus	*oryzae*	*Flavi*	genome.jgi.doe.gov/Aspor1	[12]
Aspergillus	*terreus*	*Terrei*	genome.jgi.doe.gov/Aspte1	[88]
Penicillium	*digitatum*	*Penicillium*	genome.jgi.doe.gov/Pendi1	[90]
Neurospora	*crassa*	*Neurospora*	genome.jgi.doe.gov/Neucr2	[91]
Aspergillus	*albertensis*	*Flavi*	genome.jgi.doe.gov/Aspalbe1	This study
Aspergillus	*alliaceus*	*Flavi*	genome.jgi.doe.gov/Aspalli1	This study
Aspergillus	*arachidicola*	*Flavi*	genome.jgi.doe.gov/Aspara19utr	This study
Aspergillus	*parasiticus*	*Flavi*	genome.jgi.doe.gov/Asppar1	This study
Aspergillus	*novoparasiticus*	*Flavi*	genome.jgi.doe.gov/Aspnovo1	This study
Aspergillus	*minisclerotigenes*	*Flavi*	genome.jgi.doe.gov/Aspmin1	This study
Aspergillus	*nomius*	*Flavi*	genome.jgi.doe.gov/Aspnom1	This study
Aspergillus	*pseudotamarii*	*Flavi*	genome.jgi.doe.gov/Asppset1	This study
Aspergillus	*sergii*	*Flavi*	genome.jgi.doe.gov/Aspser1	This study
Aspergillus	*tamarii*	*Flavi*	genome.jgi.doe.gov/Asptam1	This study
Aspergillus	*transmontanensis*	*Flavi*	genome.jgi.doe.gov/Asptra1	This study
Aspergillus	*leporis*	*Flavi*	genome.jgi.doe.gov/Asplep1	This study
Aspergillus	*aflatoxiniformans*	*Flavi*	genome.jgi.doe.gov/Asppari1	This study
Aspergillus	*luteovirescens (formerly bombycis)*	*Flavi*	genome.jgi.doe.gov/Aspbom1	[14]
Aspergillus	*sojae*	*Flavi*	-	[3, 24-25]

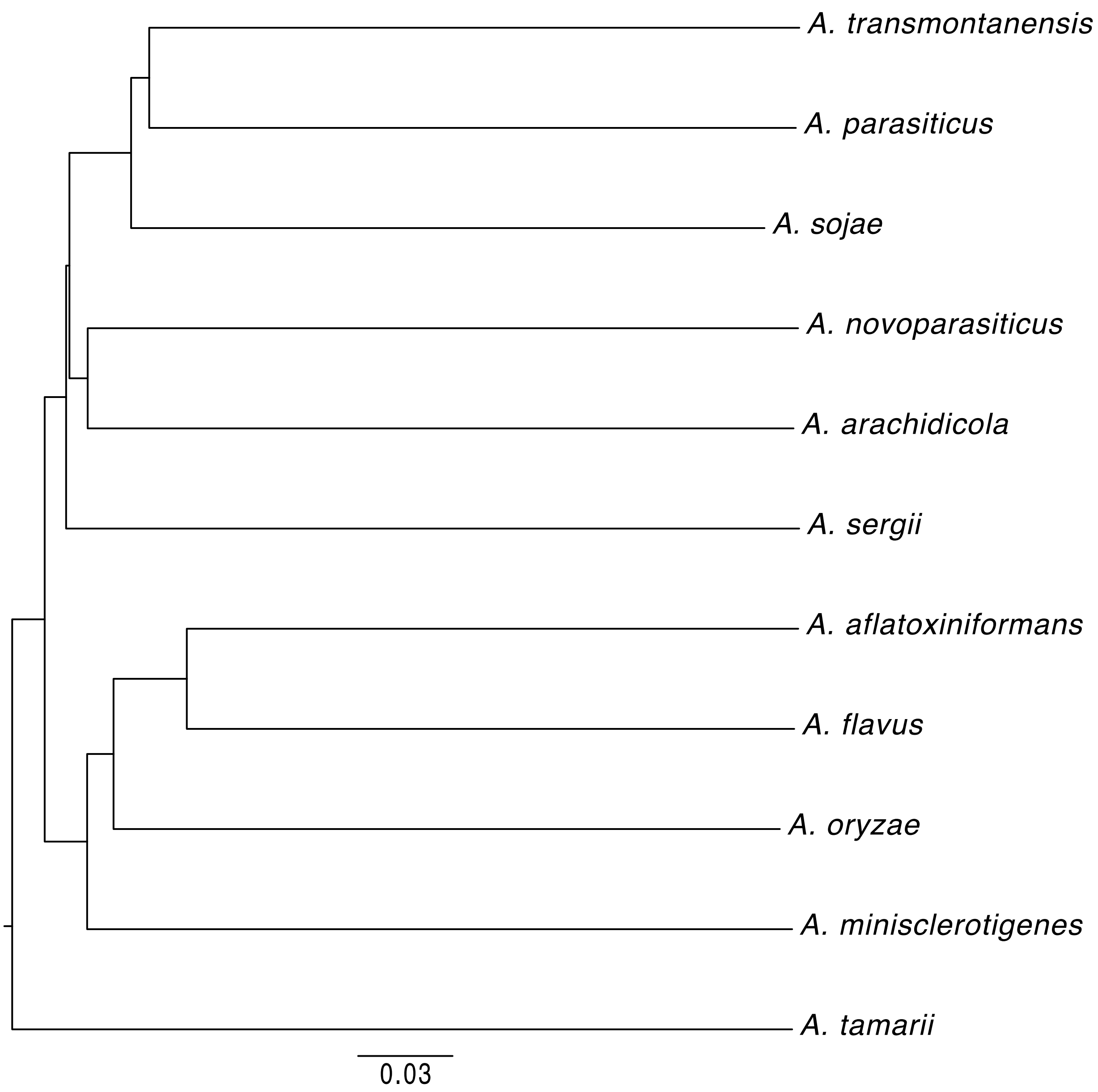

Supplementary Figure 1 - Phylogenetic tree based on composition vector approach. Phylogenetic tree constructed using CVTree[26-27] making alignment free whole genome based phylogenetic trees. This is created using the whole genome and the K'mer was set to 18.

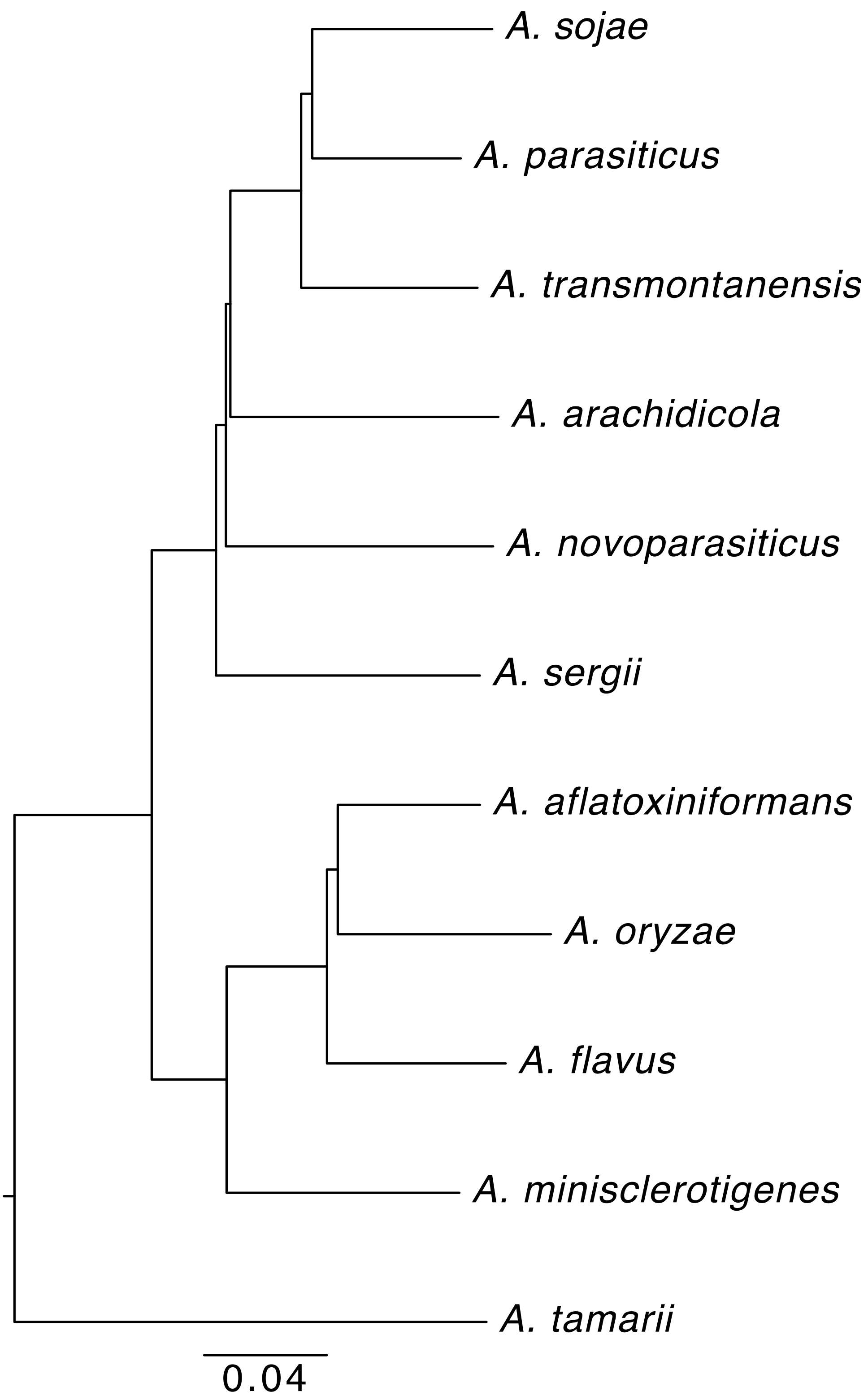

Supplementary Figure 2 Phylogenetic tree based on composition vector approach. Phylogenetic tree constructed using CVTree[26-27] making alignment free whole genome based phylogenetic trees. This is created using the whole proteome and the K'mer was set to 8.

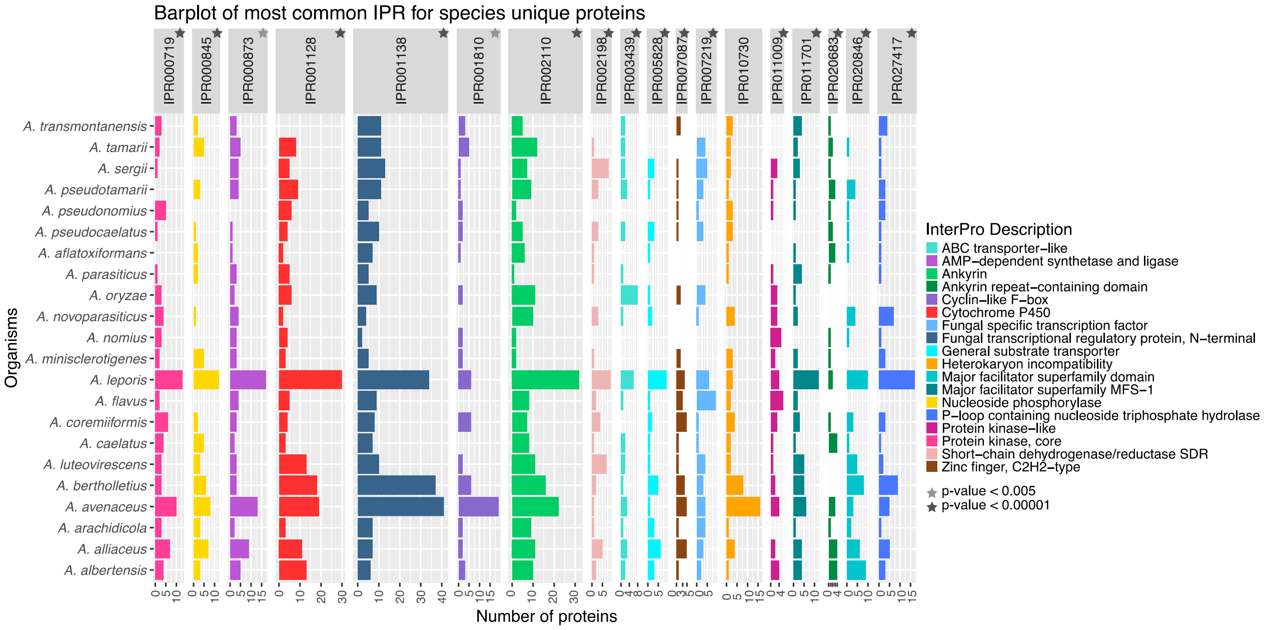

Supplementary Figure 3 - Most common InterPro domains in species unique proteins. Bar plot showing the number of species unique proteins with an InterPro domain per species, shown for the most common InterPro annotations[32]. Light grey star indicates p-values below 0.005 and dark grey star indicates p-value below 0.00001 of enrichment in the species unique genes for the specific functional domain (for both Fisher's exact and Chi-squared test).

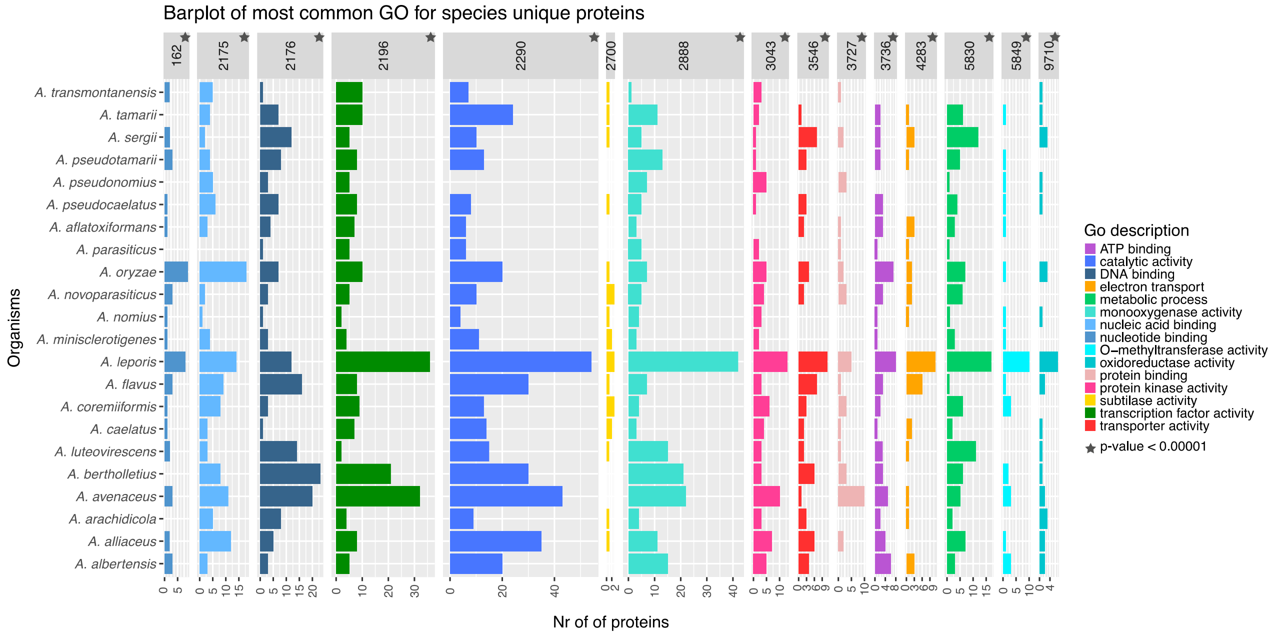

Supplementary Figure 4 - Most common GO domains in species unique proteins. Bar plot showing the number of species unique proteins with a GO domain per species, shown for the most common GO annotations[34]. Dark grey star indicates p-value < 0.00001 of enrichment in the species unique genes for the specific functional domain (for both Fisher's exact and Chi-squared test).

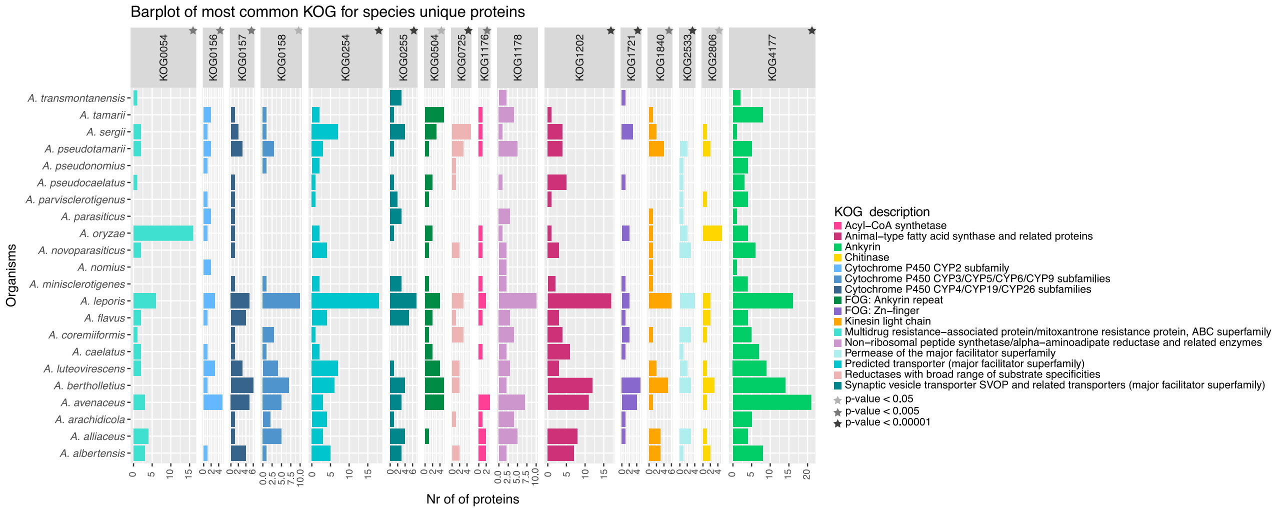

Supplementary Figure 5 - Most common KOG domains in species unique proteins. Bar plot showing the number of species unique proteins with an KOG domain per species, shown for the most common KOG annotations[35]. P-values indicates enrichment of a certain KOG term in the species unique genes for the specific functional domain. The p-values are indicated by stars of light to dark grey; the lightest star indicates p-values < 0.05, followed by p-values <0.005 and the darkest stars indicates p-value < 0.00001 (for both Fisher's exact and Chi-squared test).

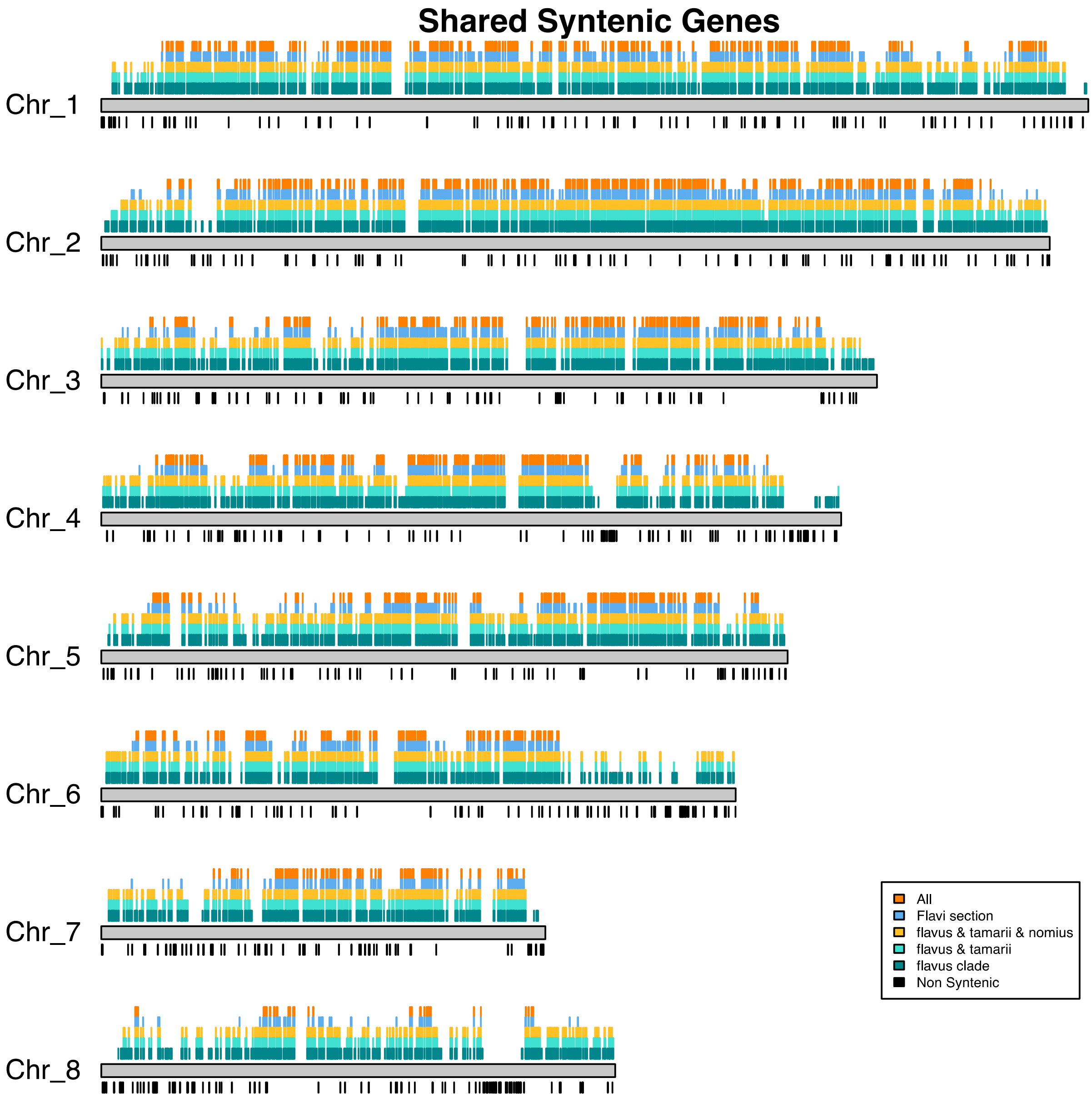

Supplementary Figure 6 - Syntenic genes mapped to the A. oryzae *genome. The grey bars represent the* A. oryzae *genome. Syntenic genes were found using* A. oryzae *as reference comparing all the other species to this genome. Above the chromosomes syntenic genes shared with all species in a certain group is shown. Dark turquoise -* A. flavus *clade; light turquoise -* A. flavus *and* A. tamarii *clade; yellow -* A. flavus, A. tamarii *and* A. nomius *clade; blue -* Flavi *section; orange -* Flavi *section*, A. nidulans *and* A. fumigatus. *Below the chromosome the non-syntenic genes, found to be non-syntenic in all the species, are shown in black.*

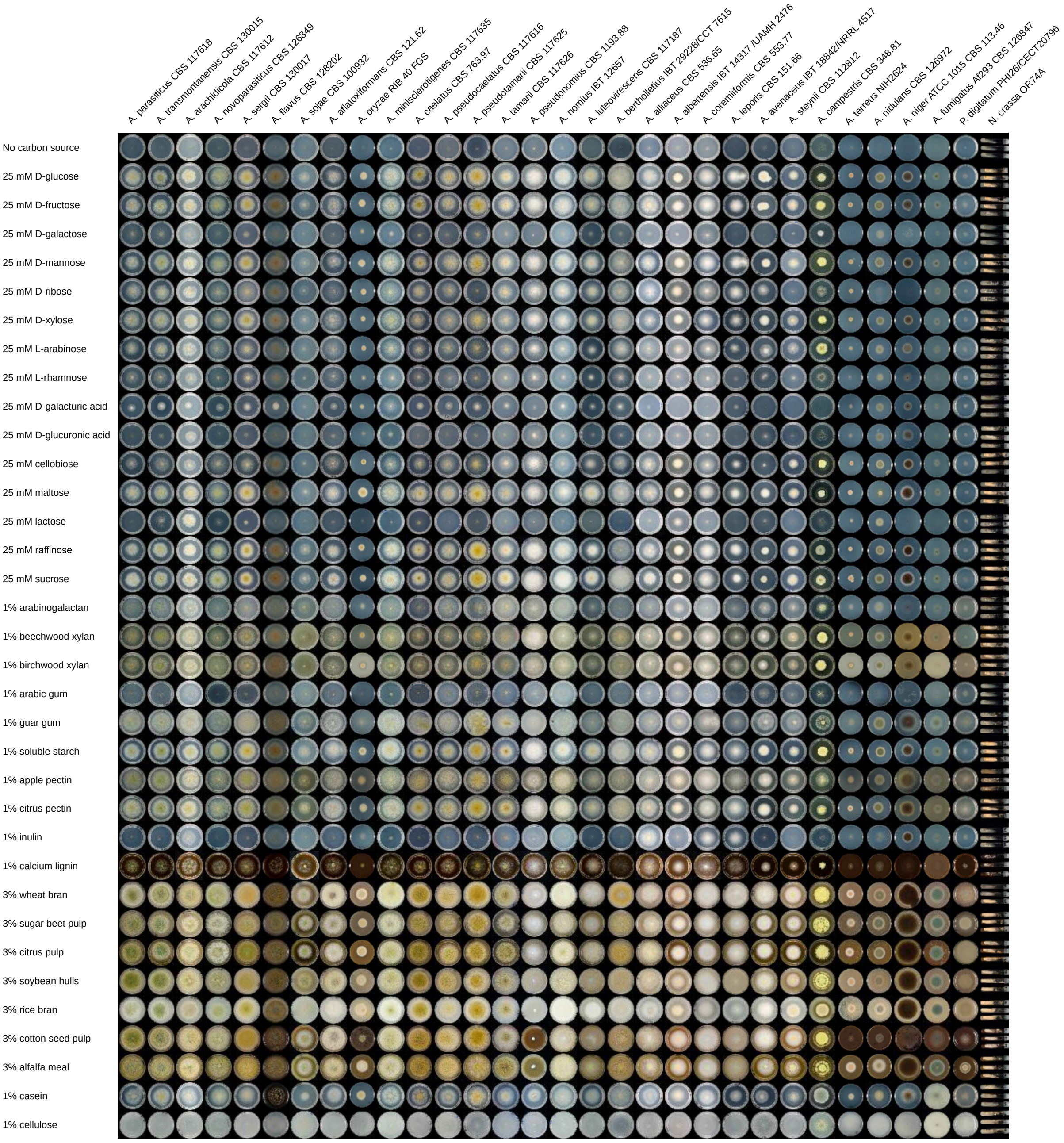

Supplementary Figure 7 - Growth analysis of 23 Flavi *species plus 8 additional species on 35 different growth media.*

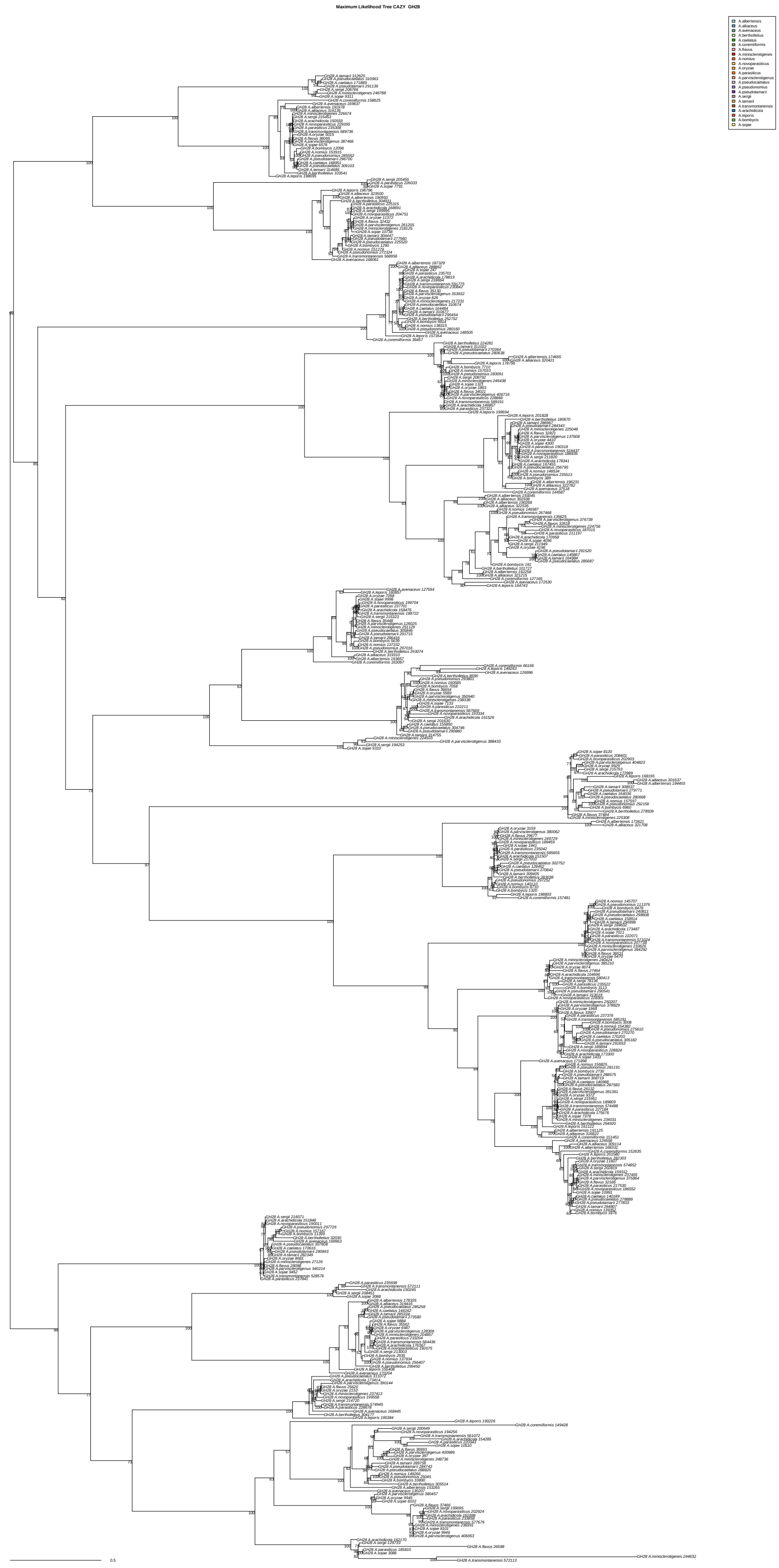

Supplementary Figure 8 - Phylogenetic tree of GH28. Phylogenetic tree of all proteins assigned to the GH28 CAZyme family. The GH28 family consists of polygalacturonase. Alignment of the members of GH28 CAZyme family found in all section Flavi species was created using clustalo. The ML phylogenetic tree was created using the ape package in R[92].

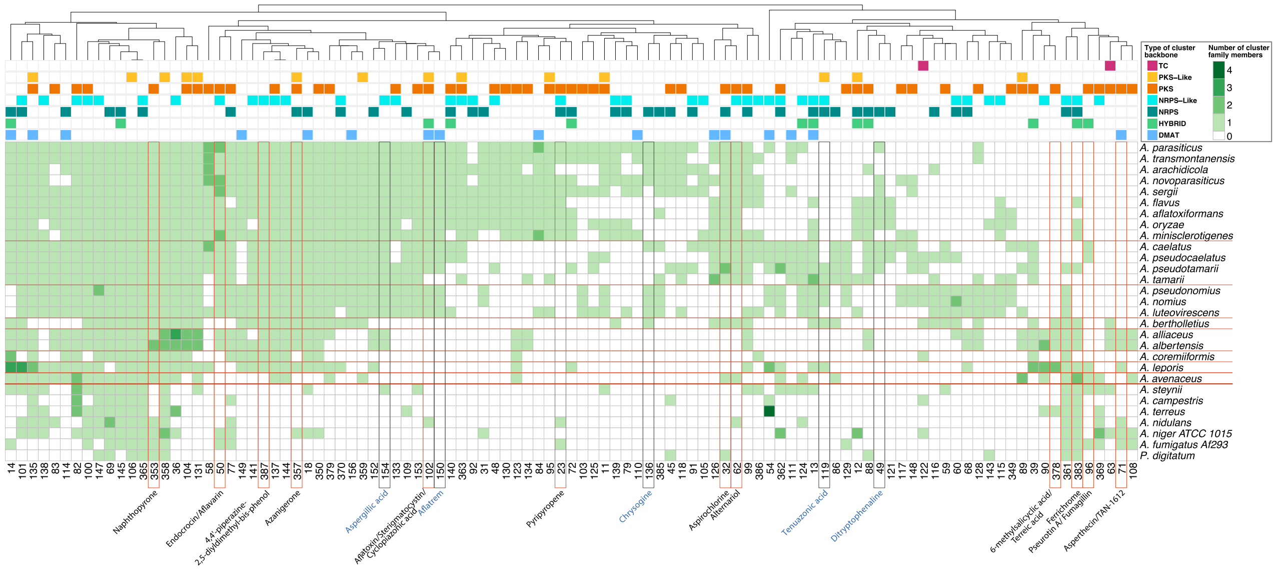

Supplementary Figure 9 - Heatmap of secondary metabolite cluster families. SM cluster families with members in at least five species are illustrated by a heatmap. The top rows indicate the backbone enzymes found within the SM cluster family. Compounds with similar SM clusters are added from the dereplication using MIBiG marked by orange boxes and black text in addition to manually curated compounds (marked by black boxes and blue text). Aspergillic acid[93], aflatrem[94], chrysogine[95], tenuazonic acid[96], ditryptophenaline[97].

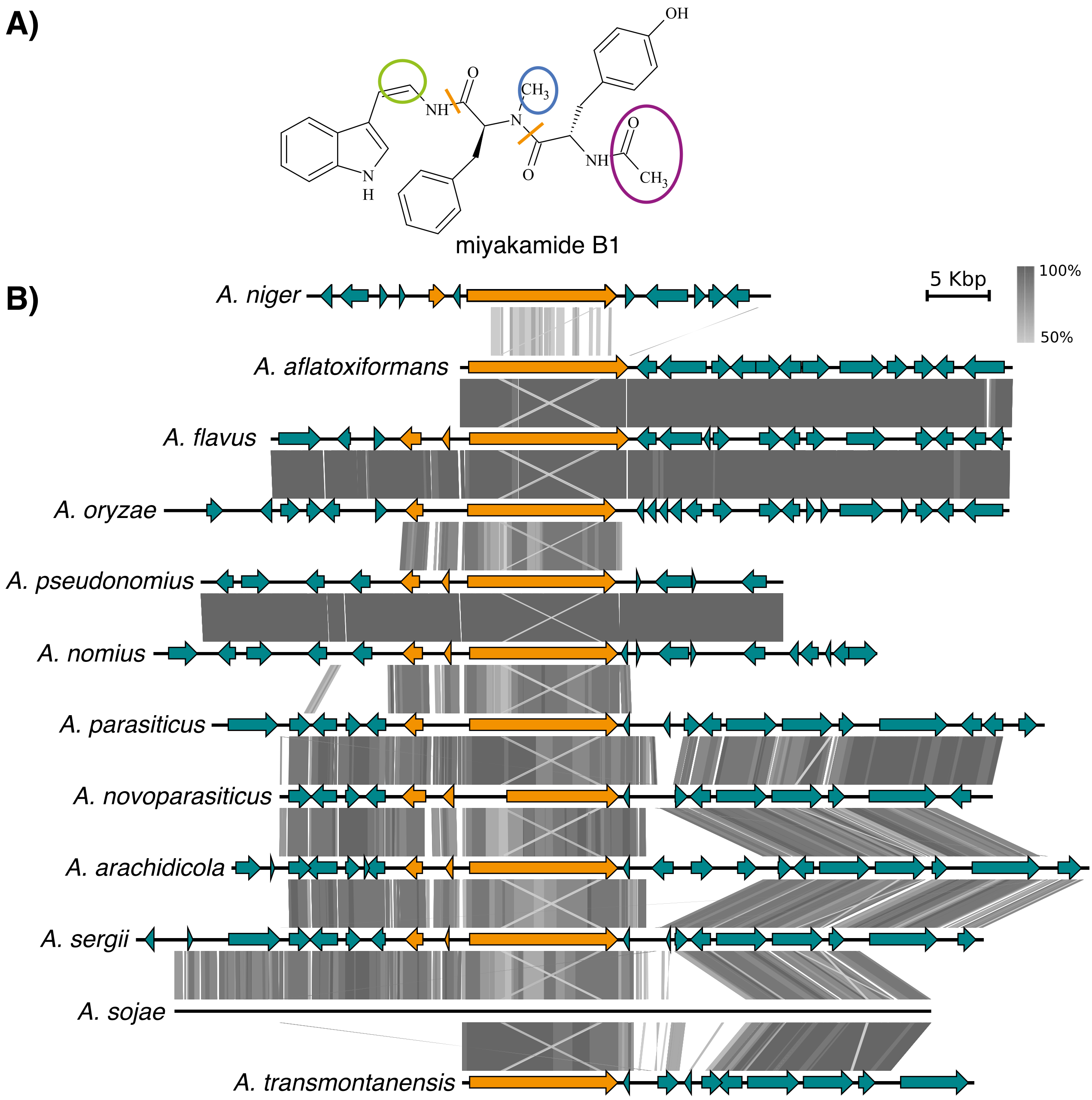

Supplementary Figure 10 - Miyakamide and putative clusters. A) Miyakamide B1 showing the three amino acid parts (organge line), the acetylation (purple circle), the decarboxylation (green circle) and the N-methylation (blue circle). B) Syntenic plot of the putative miyakamide cluster family plus surrounding genes. The syntney plot was generated using EasyFig[86] with minimum length set to 50 bp and minimum identity to 50%. The genes potentially involved in miyakamide production are marked by orange.

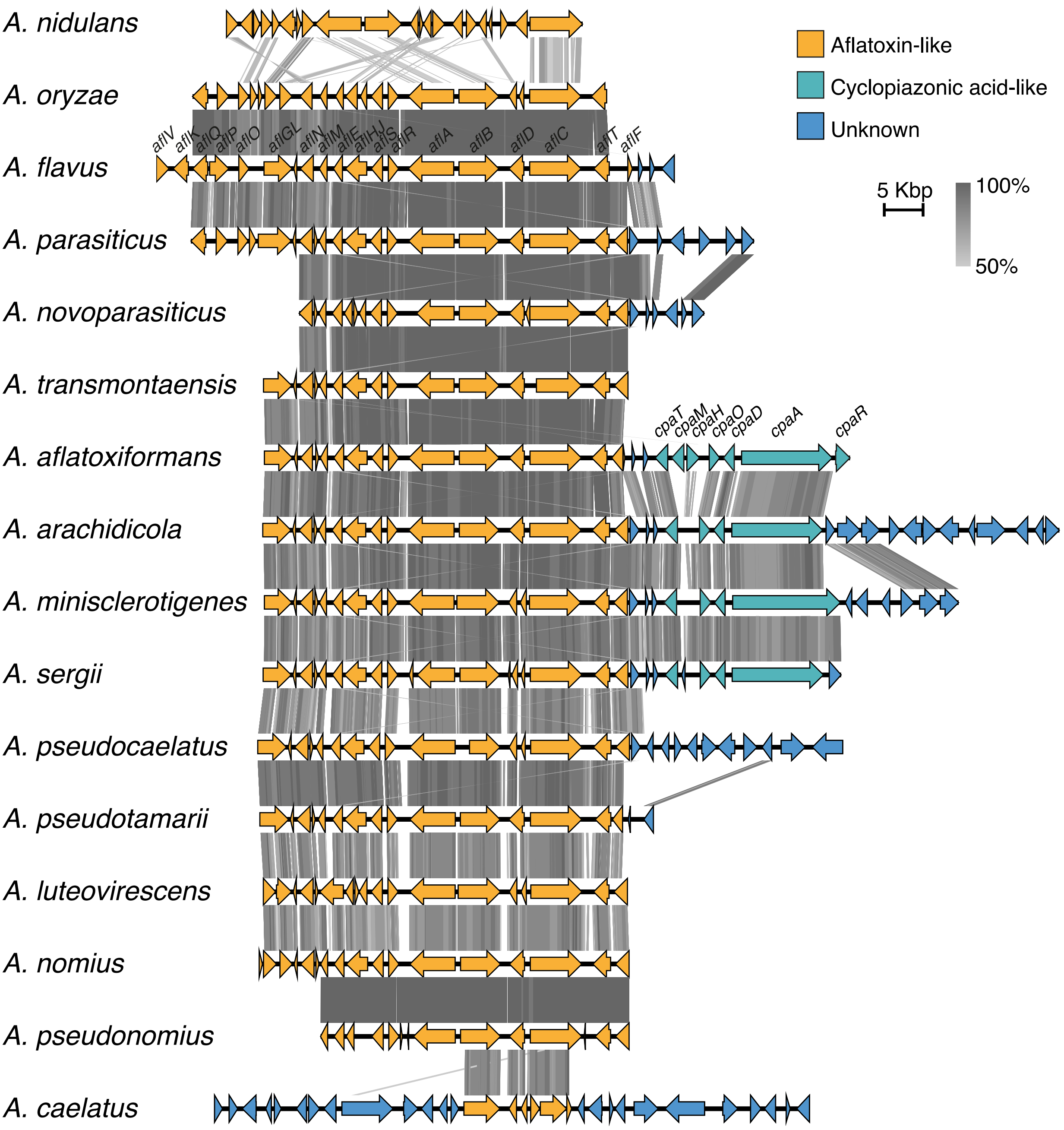

Supplementary Figure 11- Synteny of aflatoxin cluster family. Syntenic plot of the predicted clusters belonging to the 'sterigmatocystin - aflatoxin - cyclopiazonic-acid' cluster family. The synteny plot was generated using EasyFig[86] with minimum length set to 50 bp and minimum identity to 50%. The genes are color coded based on known pathways; aflatoxin (orange), cyclopiazonic-acid (turquoise) and unknown (blue).

```
CLUSTAL O(1.2.4) multiple sequence alignment


Aspfl1_36747            mlcfrsvdglstslaevsrlpssllvlsrilqickskdattvrtrlnrhtrhgplssfhs    60
Aspbom1_5312            ------------------------------------------------------------    0
Asppsec1_279719         ------------------------------------------------------------    0
Asppset1_276010         ------------------------------------------------------------    0
AsojaeX_7236            ------------------------------------------------------------    0
Aspnom13137_1_6258      ------------------------------------------------------------    0
Aspnom1_129446          ------------------------------------------------------------    0
Asppsen1_274161         ------------------------------------------------------------    0
Aspser1_195135          ------------------------------------------------------------    0
Aspmin1_217119          ------------------------------------------------------------    0
Asptra1_544984          ------------------------------------------------------------    0
Aspor1_5677             ------------------------------------------------------------    0
Asppari1_400163         ------------------------------------------------------------    0
Aspara19utr_161418      ------------------------------------------------------------    0
Asppar1_236245          ------------------------------------------------------------    0
Aspnovo1_191948         ------------------------------------------------------------    0


Aspfl1_36747            iepirpspikmtlpnkaalvglahtlseqvkrylvtadetkspedhklciegertpsste    120
Aspbom1_5312            ----------malpnktalvglanalseqvksylattdgtelskdq-----svgspsste    45
Asppsec1_279719         ----------maqpnkaalvglantlselvkryvgtadetklsedhthcmesvrspsste    50
Asppset1_276010         ----------maqpnkaalvglantlseqvkrylgtadgaklsevdthcmdgvrspsste    50
AsojaeX_7236            ----------malpk-aalvgla-tlseqvky---ltaetkspedhklcieet---psse    42
Aspnom13137_1_6258      ----------malpdkaalvglanalseqvkrylaipdetelpkgdkhciegvkssssae    50
Aspnom1_129446          ----------malpdkaalvglanalseqvkrylaiadetdlpnghkhciegannpssae    50
Asppsen1_274161         ----------malpdkaalvglanalseqvkrylaiadetdlpnghkhciegannpssae    50
Aspser1_195135          ----------malpnkaalmglantlseqvrrylatadetkspedhklciesvrspsste    50
Aspmin1_217119          ----------malpnkaalvglahtlsdqvkrylvtaddtkspedhklcierertpsste    50
Asptra1_544984          ----------malpnkaalvglantlseqvkcylatagetkspedhkvcieserapsste    50
Aspor1_5677             ----------malpnkaalvglahtlseqvkhylvtadetkspedhklciereitpsste    50
Asppari1_400163         ----------malpnkaalvglahtlseqvkhylvtadetkspedhklciereitpsste    50
Aspara19utr_161418      ----------malpnkaalvglantlseqvqryqatagetkspedhklciesertps-te    49
Asppar1_236245          ----------malpskaalvglantlseqvkrylatagetkspedhklciesertpssne    50
Aspnovo1_191948         ----------malpnkaallglantlseqvkrylatagetkspedhklciesernsssste    50
                                  *: *. :**:*** :**: *:        :.  : .             *

Aspfl1_36747            haqaweivrtcdrigslvhgpvpwllsnalshldsaclaaatqlnlqdiivdgpsptsld    180
Aspbom1_5312            haqaweivrtcdrigslihgpvpwllsnalshldsaclaaavqlnlqdiivdgpsptsld    105
Asppsec1_279719         yaqaweivrtcdrisslihgpvpwllsnalshldsaclaaatqlnlqdiivdgpsptslk    110
Asppset1_276010         haqaweivrtcdrisslihgpvpwllsnalshldsaclaaatqlnlqdiivdgpsptslk    110
AsojaeX_7236            haqaweivrtcdrigslvhgpvpwllsnalshldsaclaaa-tlnlqdiivdgpsptsld    101
Aspnom13137_1_6258      haqaweivrtcdrigslihgpvpwllsnalshldsaclaaatqlnlqdvivdgasstsld    110
Aspnom1_129446          haqaweivrtcdrigslihgpvpwllsnalshldsaclaaatqlnlqdvivdgasptsld    110
Asppsen1_274161         haqaweivrtcdrigslihgpvpwllsnalshldsaclaaatqlnlqdvivdgasptsld    110
Aspser1_195135          haqaweivrtcdrigslvhgpvpwllsnalshldsaclaaatqlnlqdiivdgpsptsld    110
Aspmin1_217119          haqaweivrtcdrigslvhgpvpwllsnalshldsaclaaatqlnlqdiivdgpspasld    110
Asptra1_544984          haqaweivrtcdrigslvhgpvpwllsnalshldsaclaaathlnlqdiivdgpsptsld    110
Aspor1_5677             haqaweivrtcdrigslvhgpvpwllsnalshldsaclaaatqlnlqdiivdgpsptsld    110
Asppari1_400163         haqaweivrtcdrigslvhgpvpwllsnalshldsaclaaatqlnlqdiivdgpsptsld    110
Aspara19utr_161418      haqaweivrtcdrigslvhgpvpwllsnalshldsaclaaathlnlqdiivdgpsptsld    109
Asppar1_236245          haqaweivrtcdrigslvhgpvpwllsnalshldsaclaaathlnlqdiivdgpsptsld    110
Aspnovo1_191948         haqaweivrtcdrigslvhgpvpwllsnalshldsaclaaathlnlqdiivdgpsptsld    110
                        :*************.**:***********************  *****:**** * :**.

Aspfl1_36747            tivtatgvsedll-----------------------------------------------    193
Aspbom1_5312            tivvatgvsedllrrilrgcaqrfifeevapgqyahtdaskmlrvtgihalvgfsrphqa    165
Asppsec1_279719         tivaatgvsedllrrilrgcaqrfifeevaadqfahtdaskmlcvtgihalvgfscpdqr    170
Asppset1_276010         tivaatgvsedllrrilrgcaqrlifeevaadqyahtdaskmlcvtgihalvgfscphqr    170
AsojaeX_7236            tivaatgvsedllrrilrgcaqrfifeevapdqyahtdaskmlrvtgihalvgfsc-hqg    160
Aspnom13137_1_6258      tivaatgvskdllrrilrgcaqrfifeevapdqyahtdaskmlrvkgihalvgfs-----    165
Aspnom1_129446          tivaatgvskdllrrilrgcaqrfifeevapdqyahtdasemlrvkgihalvgfs-----    165
Asppsen1_274161         tivaatgvskdllrrilrgcaqrfifeevapdqyahtdaskmlrvkgihalvgfs-----    165

Aspser1_195135          tivaatgvsddllrrilrgcaqrfifeevapdqyahtdaskmlrvtgihalvgfscphqg    170
Aspmin1_217119          tivaatgvsedllrrilrgcaqrfifeevapdqyahtdaskmlrvtgihalvgfsc----    166
Asptra1_544984          tivaatgvsedllrrilrgcaqrfifeevapdqyahtdaskmlrvtgihalvgfsc----    166
Aspor1_5677             tivaatgvsedllrrilrgcaqrfifeevapdqyahtdaskmlrvtgihalvgfscphqg    170
Asppari1_400163         tivaatgvsedllrrilrgcaqrfifeevapdqyahtdaskmlrvtgihalvgfscphqg    170
```

```
Aspara19utr_161418      tivaatgvsedllrrilrgcaqrfifeevapdqyahtdaskmlrvrgihalvgfscphqg    169
Asppar1_236245          tivaatgvsedllrrilrgcaqrfifeevapdqyahtdaskmlrvtgihalvgfsc----    166
Aspnovo1_191948         tivaatgvsedllrrilrgcaqrfifeevapdqyahtdaskmlrvtgihalvgfscphqg    170
                        ***.*****.***

Aspfl1_36747            ----------------------------------------ptkglfdyystvdevrgrrf    213
Aspbom1_5312            yrpscdevmrsgayfseflqqtkgnppswnvpspfslafdpakglfdyyntvdevrgrrf    225
Asppsec1_279719         drvscdevmrsgacfsdflqqtkgnppswnvpspfslafdpakglfdyysmvdevrgrrf    230
Asppset1_276010         drvscdevmhsgacfsdflqqtkgnppswnvpspfslafdpakglfdyysmvdesrgrrf    230
AsojaeX_7236            drvrcdevmrsgayfsdflqqtkgkppswnvpspfslafdptkglfdyystvdevrgrrf    220
Aspnom13137_1_6258      ----cdevmrsgacfsdflqqtkgnppswnvpspfslafdpakglfdyystvdevrgrrf    221
Aspnom1_129446          ----cdevmrsgacfsdflqqtkgnppswnvpspfslafdpakglfdyystvdevrgrrf    221
Asppsen1_274161         ----cdevmrsgacfsdflqqtkgnppswnvpspfslafdpakglfdyystvdevrgrrf    221
Aspser1_195135          drvscdevmrsgacfsdflqqtkgnpprwnvpspfslafdptkglfdyystvdevrgrrf    230
Aspmin1_217119          -----devmrsgayfsdflqqtkdnppswnvpspfslafdptkglfdyystvdevrgrrf    221
Asptra1_544984          -----devmrsgayfadflqqtkgkppswnvpspfslafdptkglfdyystvdevrgrrf    221
Aspor1_5677             drvrcdevmrsgayfsdflqqtkgkppswnvpspfslafdptkglfdyystvdevrgrrf    230
Asppari1_400163         drvrcdevmrsgayfsdflqqtkgkppswnvpspfslafdptkglfdyystvdevrgrrf    230
Aspara19utr_161418      drvrcdevmrsgayfsdflqqtkgkppswnvpspfslafdptkglfdyystvdevrgrrf    229
Asppar1_236245          -----devmrsgasfsdflqqtkgkppswnvpspfslafdptkglfdyystvdevrgrrf    221
Aspnovo1_191948         drvrcdevmrsgayfsdflqqtkgkppswnvpspfslafdptkglfdyystvdevrgrrf    230
                                                                *:*******. *** *****

Aspfl1_36747            dlgmggteatkplveemfdfsslpegstvvdvgggrghlsrrvsqkhphlrfivqdlpav    273
Aspbom1_5312            dlgmggteatkplveemfdfnslpegstvvdvgggrghlsrrvsqkhsqlkfivqdlpav    285
Asppsec1_279719         dlgmggteatkplveemfdfsslpegstvvdvgggrghlsrrvsqkyphlrfivqdlpav    290
Asppset1_276010         dlgmggteatkplveemfdfsslpegstvvdigggrghlsrrvsqkyphlrfivqdlpav    290
AsojaeX_7236            dlgmggteatkplveemfdfsslpegstvvdvgggrghlsrrvsqkhphlrfivqdlpav    280
Aspnom13137_1_6258      dlgmggteatkplveemfdfsslsegstivdvgggrghlsrrvsqkhphlkfivqdlpav    281
Aspnom1_129446          dlgmggteatkplveemfdfsslpegstvvdvgggrghlsrrvsqkhphlkfivqdlpav    281
Asppsen1_274161         dlgmggteatkplveemfdfsslpegstvvdvgggrghlsrrvsqkhphlkfivqdlpav    281
Aspser1_195135          dlgmggteatkplveemfdfsslpegstvvdvgggrghlsrrvsqkhphlkfivqdlpav    290
Aspmin1_217119          dlgmggteatkplveemfdfsslpegstvvdvgggrghlsrrvsqkhphlkfivqdlpav    281
Asptra1_544984          dlgmggteatkplveemfdfsslpeestvvdvgggrghlsrrvsqkhphlrfivqdlpav    281
Aspor1_5677             dlgmggteatkplveemfdfsslpegstvvdvgggrghlsrrvsqkhphlrfivqdlpav    290
Asppari1_400163         dlgmggteatkplveemfdfsslpegstvvdvgggrghlsrrvsqkhphlrfivqdlpav    290
Aspara19utr_161418      dlgmggteatkplveemfdfsslpegstvvdvgggrghlsrrvsqkhphlrfivqdlpav    289
Asppar1_236245          dlgmggteatkplveemfdfsslpegstvvdvgggrghlsrrvsqkhphlrfivqdlpav    281
Aspnovo1_191948         dlgmggteatkplveemfdfsslpegsavvdvgggrghlsrrvsqkhphlrfivqdlpav    290
                        ********************.** * *::**:**************: :*:*********

Aspfl1_36747            ihgvedtdkvtmmehdirrrnpvrgadvyllrsilhdypdaacveilsnivtamdpsksr    333
Aspbom1_5312            iqgvedtdqvtmmehdirhpnpvrgadvyllrsilhdypdaacveilsnivsamdpvksr    345
Asppsec1_279719         ihgvedtdkvtmmehdirhpnpvrgadvyflrsilhdypdaacveilsnivsamdptksr    350
Asppset1_276010         ihgvddtdkvtmmehdirhpnpvrgadvyflrsilhdypdaacveilsnivsamdptksr    350
AsojaeX_7236            ihgvedtdkvtmmehdirrpnpvrgadvyllrsilhdypdaacveilsnivtamdpsksr    340
Aspnom13137_1_6258      ihgvedtekvtmmehdirhpnpvrgadvyllrsilhdypdaacveilsnivsamdptksr    341
Aspnom1_129446          ihgvedtekvtmmehdirhpnpvrgadvyllrsilhdypdaacveilsnivsamdptksr    341
Asppsen1_274161         ihgvedtekvtmmehdirhpnpvrgadvyllrsilhdypdaacveilsnivsamdptksr    341
Aspser1_195135          ihgvedtdkvtmvehdirhpnpvrgadvyllrsilhdypdaacveilsnivsamdpiksr    350
Aspmin1_217119          icgvedtdkvtmmehdirhpnpvrgadvyllrsilhdypdaacveilsnivsamdpiksr    341
Asptra1_544984          ihgvedtdkvtmmehdirhqnpvrgadvyllrsilhdypdaacveilsnivsamdptksr    341
Aspor1_5677             ihgvedtdkvtmmehdirrpnpvrgadvyllrsilhdypdaacveilsnivtamdpsksr    350
Asppari1_400163         ihgvedtdkvtmmehdirrpnpvrgadvyllrsilhdypdaacveilsnivtamdpsksr    350
Aspara19utr_161418      ihgvedtdkvtmmehdirrpnpmrgadvyllrsilhdypdaacveilsnivtamdpsksr    349
Asppar1_236245          ihgvedtdkvtmmehdirrpnpvrgadvyllrsilhdypdaacveilsnivtamdpsksr    341
Aspnovo1_191948         ihgvedtdkvtmmehdirrpnpvrgadvyllrsilhdypdaacveilsnivtamdpsksr    350
                        * **:**::***:*****: **:******:*********************:**** ***

Aspfl1_36747            illdemimpdllaqdsqrfmnqidmtvvltlngkerstkewnslitmvdnrletekiwwr    393
Aspbom1_5312            illdemvmpdllaqdsqrfmnqidmtvvltlngkerstkewdsliatvegglktektwwr    405
Asppsec1_279719         illdemvvpdllaqdsqrfmnqidmtvvltlngkerstkewdsliatvgggleiektwwr    410
Asppset1_276010         illdemvvpdilaqdsqrfmnqidmtvvltlngkerstkewdslitavdggleiektwwr    410
AsojaeX_7236            illdemimpdl-aqdsqrfmnqidmtvvltlngkers-kewnsli-tvdgrletekiwwr    397
Aspnom13137_1_6258      illdemivpdllaqdsqrfmnqidmtvvltlngkersvkewdslittvdgkletektwwr    401
Aspnom1_129446          illdemvvpdllaqdsqrfmnqidmtvvltlngkersakewdslittvdgkletektwwr    401
Asppsen1_274161         illdemvvpdllaqdsqrfmnqidmtvvltlngkersakewdslittvdgkletektwwr    401
Aspser1_195135          illdemimpdflaqhsqrfmnqidmtvvltlngkerstkewdsliktvdgrletektwwr    410
Aspmin1_217119          illdemimpdllaqdsqrfmnqidmtvvltlngkerstkewdslittvdarletektwwr    401
Asptra1_544984          illdemimpdllaqdsqrfmnqidmtvvltlngkerstkewdslitavdgkletekiwwr    401
Aspor1_5677             illdemimpdllaqdsqrfmnqidmtvvltlngkerspkewnslitmvdgrletekiwwr    410
```

```
Asppari1_400163        illdemimpdllaqdsqrfmnqidmtvvltlngkerspkewnslitmvdgrletekiwwr    410
Aspara19utr_161418     illdemimpdllaqdsqrfmnqidmtvvltlngkerstkewnslittvdgrletekiwwr    409
Asppar1_236245         illdemimpdllaqdsqrfmnqidmtvvltlngkerstkewnslittvdgrletekiwwr    401
Aspnovo1_191948        illdemimpdllaqdsqrfmnqidmtvvltlngkerstkewnslittvdgrletekiwwr    410
                       ******::**: **.********************* ***:***  *   *: ** ***

Aspfl1_36747           kgeegshwgvqqlrlrk*----  410
Aspbom1_5312           qgeegshwgvqqlrlrgnsak*  426
Asppsec1_279719        kgeegshwgvqqlrlrgnsak*  431
Asppset1_276010        kgeegshwgvqqlrlrgnsak*  431
AsojaeX_7236           kgeegshwgvqqlrlrk-----  414
Aspnom13137_1_6258     kgeegshwgvqqlrlhgssad*  422
Aspnom1_129446         kgeegshwgvqqlrlhgslan*  422
Asppsen1_274161        kgeegshwgvqqlrlhgslan*  422
Aspser1_195135         kgeegshwgvqqlrlrk*----  427
Aspmin1_217119         kgeegshwgvqqlrlrk*----  418
Asptra1_544984         kgeegshwgvqqlrlrk*----  418
Aspor1_5677            kgeegshwgvqqlrlrk*----  427
Asppari1_400163        kgeegshwgvqqlrlrk*----  427
Aspara19utr_161418     kgeegshwgvqqlrlrk*----  426
Asppar1_236245         kgeegshwgvqqlrlrk*----  418
Aspnovo1_191948        kgeegshwgvqqlrlrk*----  427
                       :**************:
```


Supplementary Figure 12 - Investigation of aflP and aflQ. The aflP gene is important for the last steps in the biosynthesis of aflatoxin, but it is missing in most of the predicted aflatoxin clusters. Here is an alignment of the AflP protein against the best hits in the other Flavi *species.*

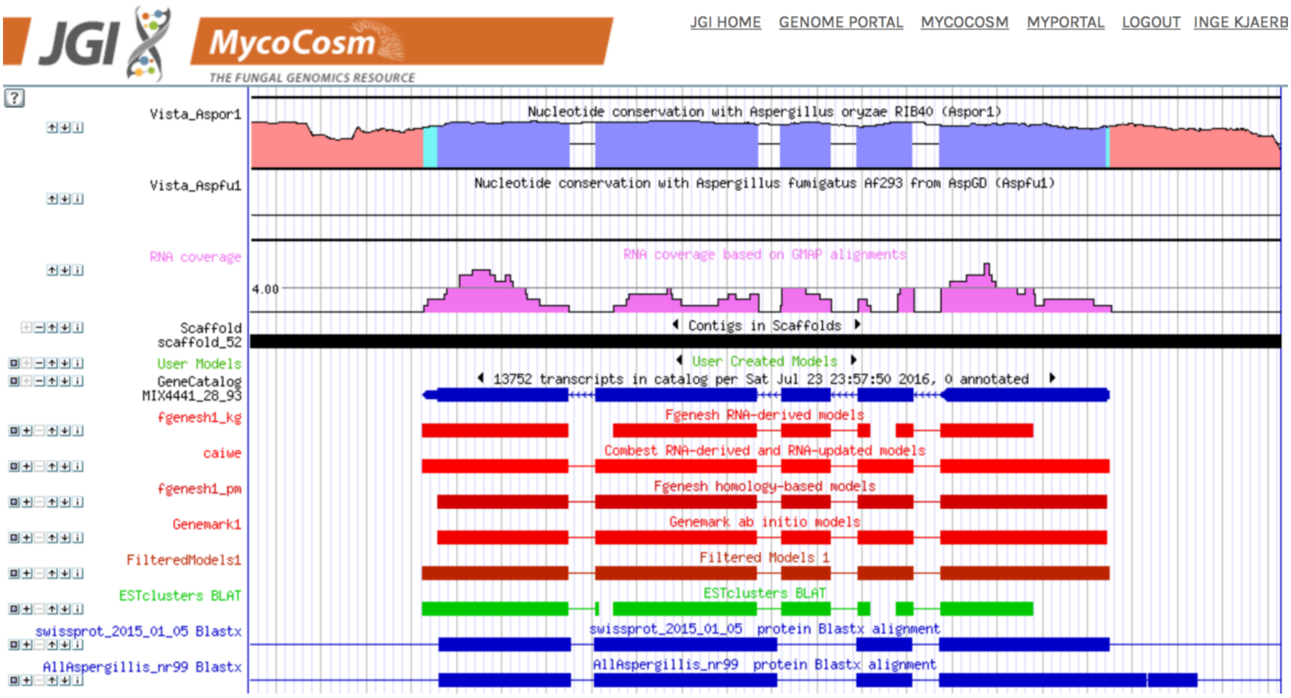

Supplementary Figure 13 - Gene prediction of aflP. An overview of the gene prediction in the aflP *gene in* A. parasiticus *and the RNA coverage from JGI at Mycocosm.*

Supplementary Data

Supplementary Data 1 - Quantitative growth analysis of 23 Flavi *species plus 8 additional species on 35 different growth media, quantitated by growth from 0-10, normalized based on growth on 1% glucose.*

Supplementary Data 2 - CAZyme content in section Flavi*. Overview of the CAZyme content and plant degradation related CAZyme content.*

Supplementary Data 3 - Secondary metabolite gene clusters section Flavi*. Long format table with all the predicted clusters in the species and the cluster family they belong to. Column 1 - Species, column 2 - JGI protein id of the predicted backbone, column 3 - type of the predicted backbone, column 4 - size of the cluster, column 5 - cluster family number.*

Supplementary Data 4 - Compounds produced by Flavi *species after growth on CYA 7 days.*
