# Supplementary material for: A comparative genomics study of 23 Aspergillus species from section Flavi
Source: Nat Commun. 2020 Feb 27;11:1106. doi: 10.1038/s41467-019-14051-y (PMC7046712; doi:10.1038/s41467-019-14051-y)
Supplement: Supplementary file 3 — Description of Additional Supplementary Files [file 41467_2019_14051_MOESM3_ESM.pdf]

Description of Additional Supplementary Files

File Name: Supplementary Data 1
Description: Quantitative growth analysis of 23 Flavi species plus 8 additional species on 35 different growth media, quantitated by growth from 0-10, normalized based on growth on 1% glucose.

File Name: Supplementary Data 2
Description: CAZyme content in section Flavi. Overview of the CAZyme content and plant degradation related CAZyme content.

File Name: Supplementary Data 3
Description: Secondary metabolite gene clusters section Flavi. Long format table with all the predicted clusters in the species and the cluster family they belong to. Column 1 - Species, column 2 - JGI protein id of the predicted backbone, column 3 - type of the predicted backbone, column 4 - size of the cluster, column 5 - cluster family number.

File Name: Supplementary Data 4
Description: Compounds produced by Flavi species after growth on CYA 7 days.
