# Supplementary material for: A comparative genomics study of 23 Aspergillus species from section Flavi
Source: Nat Commun. 2020 Feb 27;11:1106. doi: 10.1038/s41467-019-14051-y (PMC7046712; doi:10.1038/s41467-019-14051-y)
Supplement: Supplementary file 8 — Reporting Summary [file 41467_2019_14051_MOESM8_ESM.pdf]

nature research

Corresponding author(s): Mikael R. Andersen

Last updated by author(s): Oct 18, 2019

Reporting Summary

Nature Research wishes to improve the reproducibility of the work that we publish. This form provides structure for consistency and transparency in reporting. For further information on Nature Research policies, see Authors & Referees and the Editorial Policy Checklist.

Statistics

For all statistical analyses, confirm that the following items are present in the figure legend, table legend, main text, or Methods section.

n/a	Confirmed	
☐	☒	The exact sample size (*n*) for each experimental group/condition, given as a discrete number and unit of measurement
☒	☐	A statement on whether measurements were taken from distinct samples or whether the same sample was measured repeatedly
☒	☐	The statistical test(s) used AND whether they are one- or two-sided<br>*Only common tests should be described solely by name; describe more complex techniques in the Methods section.*
☒	☐	A description of all covariates tested
☒	☐	A description of any assumptions or corrections, such as tests of normality and adjustment for multiple comparisons
☒	☐	A full description of the statistical parameters including central tendency (e.g. means) or other basic estimates (e.g. regression coefficient) AND variation (e.g. standard deviation) or associated estimates of uncertainty (e.g. confidence intervals)
☒	☐	For null hypothesis testing, the test statistic (e.g. *F*, *t*, *r*) with confidence intervals, effect sizes, degrees of freedom and *P* value noted<br>*Give P values as exact values whenever suitable.*
☒	☐	For Bayesian analysis, information on the choice of priors and Markov chain Monte Carlo settings
☒	☐	For hierarchical and complex designs, identification of the appropriate level for tests and full reporting of outcomes
☒	☐	Estimates of effect sizes (e.g. Cohen's *d*, Pearson's *r*), indicating how they were calculated

Our web collection on statistics for biologists contains articles on many of the points above.

Software and code

Policy information about availability of computer code

Data collection: No software was used

Data analysis: No new custom software was used.

For manuscripts utilizing custom algorithms or software that are central to the research but not yet described in published literature, software must be made available to editors/reviewers. We strongly encourage code deposition in a community repository (e.g. GitHub). See the Nature Research guidelines for submitting code & software for further information.

Data

Policy information about availability of data

All manuscripts must include a data availability statement. This statement should provide the following information, where applicable:

- Accession codes, unique identifiers, or web links for publicly available datasets
- A list of figures that have associated raw data
- A description of any restrictions on data availability

Genomes are available as:
BioProject BioSample Accession Organism

PRJNA333904 SAMN05446748 SWDY00000000 Aspergillus nomius IBT 12657
PRJNA333902 SAMN05443097 SWDZ00000000 Aspergillus minisclerotigenes CBS 117635
PRJNA333906 SAMN05446842 SWCZ00000000 Aspergillus parasiticus CBS 117618
PRJNA333905 SAMN05446749 SWDA00000000 Aspergillus novoparasiticus CBS 126849
PRJNA333901 SAMN05446746 SWBU00000000 Aspergillus leporis CBS 151.66
PRJNA334014 SAMN05446797 SWAS00000000 Aspergillus alliaceus CBS 536.65
PRJNA333907 SAMN05446750 SWAT00000000 Aspergillus parvisclerotigenus CBS 121.62

PRJNA333910	SAMN05446752	STFH00000000	Aspergillus pseudotamarii CBS 117625
PRJNA333897	SAMN05446841	STFI00000000	Aspergillus avenaceus IBT 18842
PRJNA333912	SAMN05446599	STFJ00000000	Aspergillus tamarii CBS 117626
PRJNA333913	SAMN05446753	STFK00000000	Aspergillus transmontanensis CBS 130015
PRJNA333911	SAMN05443096	STFL00000000	Aspergillus sergii CBS 130017
PRJNA333896	SAMN05446840	STFM00000000	Aspergillus arachidicola CBS 117612
PRJNA333900	SAMN05446844	STFN00000000	Aspergillus coremiiformis CBS 553.77
PRJNA333899	SAMN05445973	STFO00000000	Aspergillus caelatus CBS 763.97
PRJNA333898	SAMN05446843	STFP00000000	Aspergillus bertholletius IBT 29228
PRJNA333895	SAMN05446902	STFQ00000000	Aspergillus albertensis IBT 14317
PRJNA333909	SAMN05446751	STFR00000000	Aspergillus pseudonomius CBS 119388
PRJNA333908	SAMN05445982	STFS00000000	Aspergillus pseudocaelatus CBS 117616

Field-specific reporting

Please select the one below that is the best fit for your research. If you are not sure, read the appropriate sections before making your selection.

☒ Life sciences ☐ Behavioural & social sciences ☐ Ecological, evolutionary & environmental sciences

For a reference copy of the document with all sections, see nature.com/documents/nr-reporting-summary-flat.pdf

Life sciences study design

All studies must disclose on these points even when the disclosure is negative.

Sample size	It is not the state of the art to perform replicates of genome sequencing.
Data exclusions	No data was excluded
Replication	It is not the state of the art to perform replicates of genome sequencing. Chemical analysis was replicated.
Randomization	No randomization needed, as no statistical tests were performed
Blinding	No blinding needed, as no statistical tests were performed

Reporting for specific materials, systems and methods

We require information from authors about some types of materials, experimental systems and methods used in many studies. Here, indicate whether each material, system or method listed is relevant to your study. If you are not sure if a list item applies to your research, read the appropriate section before selecting a response.

Materials & experimental systems

n/a	Involved in the study
☒	☐ Antibodies
☒	☐ Eukaryotic cell lines
☒	☐ Palaeontology
☒	☐ Animals and other organisms
☒	☐ Human research participants
☒	☐ Clinical data

Methods

n/a	Involved in the study
☒	☐ ChIP-seq
☒	☐ Flow cytometry
☒	☐ MRI-based neuroimaging
